# Supplementary figures and images for: Extraction of radiographic findings from unstructured thoracoabdominal computed tomography reports using convolutional neural network based natural language processing
Source: PLoS One. 2020 Jul 30;15(7):e0236827. doi: 10.1371/journal.pone.0236827 (PMC7392233; doi:10.1371/journal.pone.0236827)

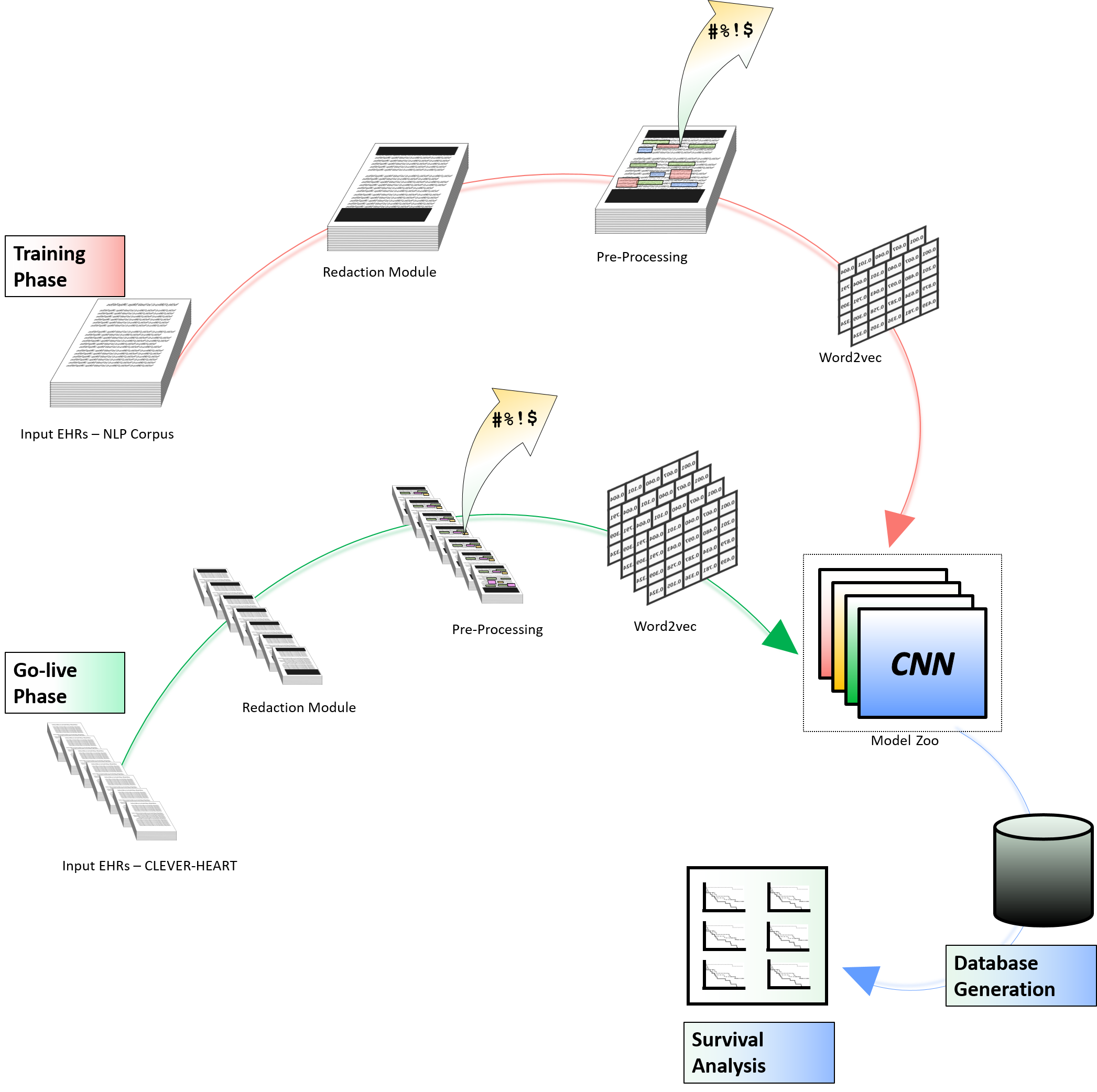

Supplement: S1 Fig — The input in the training phase consisted of 1,560 free-text thoracoabdominal computed tomography reports. These reports passed through redaction module to remove headers consisting of non-clinically relevant free text. The filtering module tokenized and removed special characters. After converting these reports into 200 dimensional vectors for each word, they were then used to train the CNN model. The output of the training phase was a trained CNN model. A separate CNN model for each of the 14 clinical findings constituted the model zoo. In the Go Live phase, input consisting of 11,808 free-text reports was passed to the trained CNN models after passing through the redaction module, the filtering module, and conversion to word vectors. The output from this phase generated the database of clinical findings which, along with age and gender, were used for prediction of all-cause mortality. (TIF) [file pone.0236827.s001.tif]

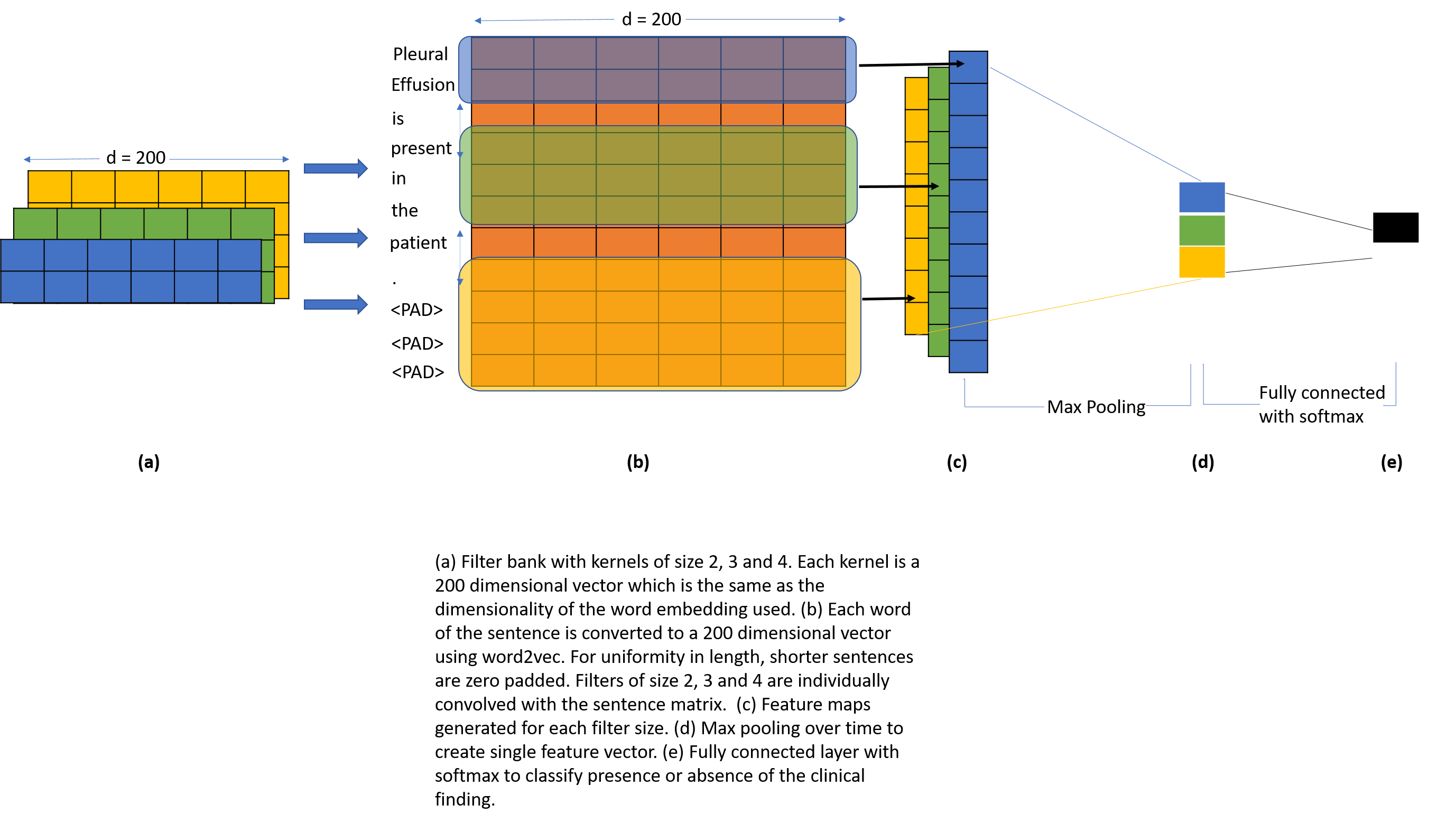

Supplement: S2 Fig — (a) Filter bank with kernels of size 2, 3 and 4. Each kernel is a 200-dimensional vector which is the same as the dimensionality of the word embedding used. (b) Each word of the sentence is converted to a 200-dimensional vector using word2vec. For uniformity in length, shorter sentences are zero padded. Filters of size 2, 3 and 4 are individually convolved with the sentence matrix. (c) Feature maps generated for each filter size. (d) Max pooling over time to create single feature vector. (e) Fully connected layer with SoftMax to classify presence or absence of the specific clinical finding. (TIF) [file pone.0236827.s002.tif]

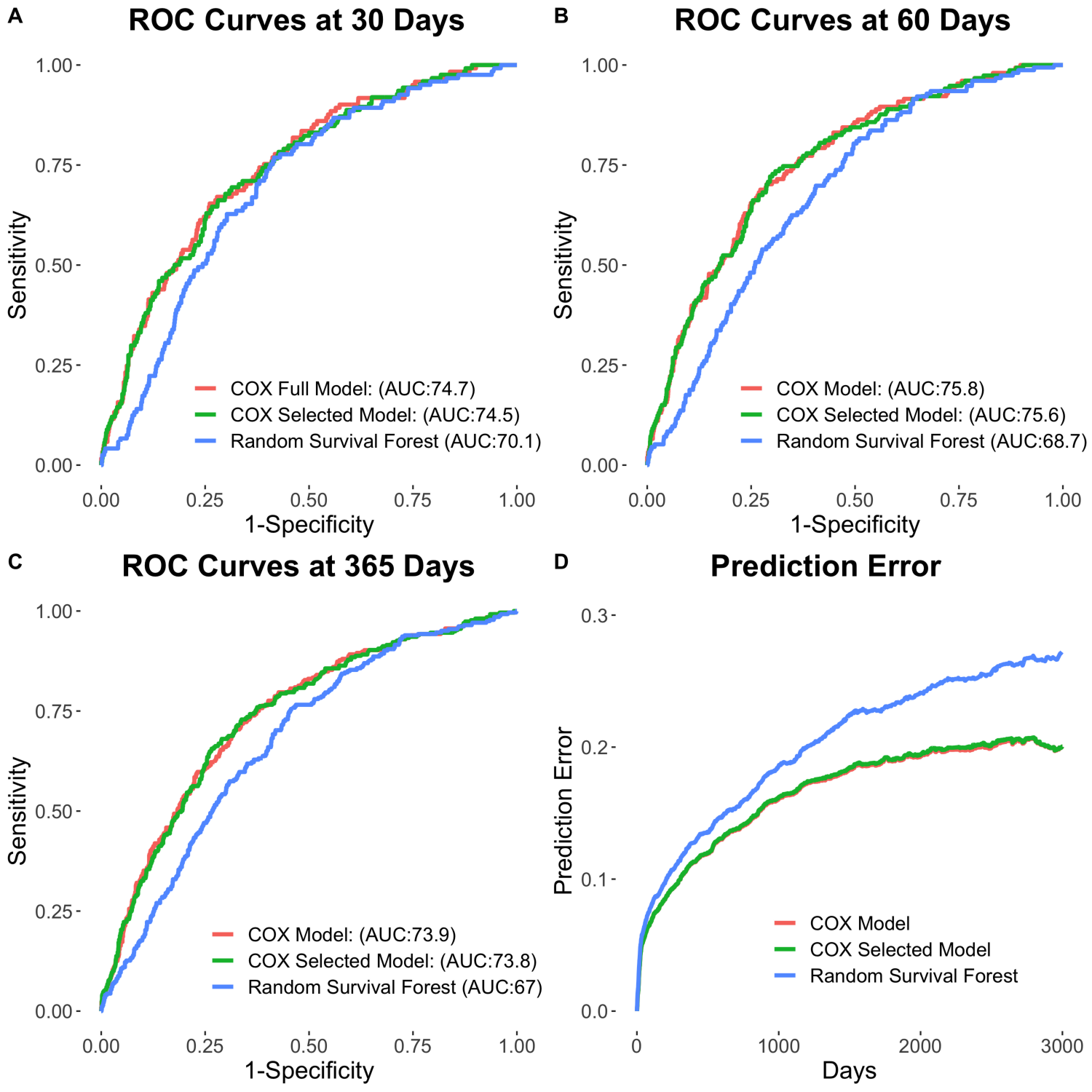

Supplement: S3 Fig — (A-C) Time-dependent ROC curves at 30, 60 and 365 days for 3 models created using the deep learning-based method for feature extraction: (1) a full COX model, (2) a COX model with select variables and (3) a random survival forest. Panel D shows the time-dependent Brier scores. (TIFF) [file pone.0236827.s003.tiff]
