# Supplementary material for: Extraction of radiographic findings from unstructured thoracoabdominal computed tomography reports using convolutional neural network based natural language processing
Source: PLoS One. 2020 Jul 30;15(7):e0236827. doi: 10.1371/journal.pone.0236827 (PMC7392233; doi:10.1371/journal.pone.0236827)
Supplement: S1 Table — (A) Full Cox model for correlation between the radiographic finding and all-cause mortality. (B) Select Cox model and correlation between radiographic findings and all-cause mortality. (DOCX) [file pone.0236827.s004.docx]

**S1 Table.** (A) Full Cox model for correlation between the radiographic finding and all-cause mortality. (B) Select Cox model and correlation between radiographic findings and all-cause mortality.

| S1 Table A | | | | | |
| --- | --- | --- | --- | --- | --- |
| No. | **Radiographic Finding** | **HR** | **Lower** | **Upper** | **P value** |
| 1 | Aortic Aneurysm | 0.97 | 0.82 | 1.14 | 0.69 |
| 2 | Ascites | 1.68 | 1.51 | 1.88 | <0.001 |
| 3 | Atelectasis | 1.02 | 0.94 | 1.11 | 0.59 |
| 4 | Atherosclerosis | 1.13 | 1.04 | 1.22 | 0.00 |
| 5 | Cardiomegaly | 1.15 | 1.05 | 1.26 | 0.00 |
| 6 | Enlarged Liver | 1.07 | 0.74 | 1.53 | 0.72 |
| 7 | GB Thickening | 0.21 | 0.03 | 1.50 | 0.12 |
| 8 | Hernia | 0.88 | 0.79 | 0.97 | 0.01 |
| 9 | Hydronephrosis | 0.98 | 0.79 | 1.38 | 0.92 |
| 10 | Lymphadenopathy | 1.16 | 1.06 | 1.27 | 0.00 |
| 11 | Pleural Effusion | 1.63 | 1.48 | 1.78 | <0.001 |
| 12 | Pneumonia | 1.33 | 1.19 | 1.48 | <0.001 |
| 13 | Previous Surgery | 1.00 | 0.92 | 1.48 | <0.001 |
| 14 | Pulmonary Edema | 1.27 | 1.05 | 1.54 | 0.01 |
| 15 | Age at Report | 1.02 | 1.02 | 1.03 | <0.001 |
| 16 | Factor (Gender) M | 1.16 | 1.07 | 1.26 | <0.001 |

| S1 Table B | | | | | |
| --- | --- | --- | --- | --- | --- |
| No. | **Radiographic Finding** | **HR** | **Lower** | **Upper** | **P value** |
| 1 | Ascites | 1.67 | 1.50 | 1.86 | <0.001 |
| 2 | Atelectasis | 1.02 | 0.94 | 1.11 | 0.63 |
| 3 | Cardiomegaly | 1.18 | 1.08 | 1.29 | <0.001 |
| 4 | Hernia | 0.87 | 0.79 | 0.96 | 0.01 |
| 5 | Lymphadenopathy | 1.17 | 1.06 | 1.28 | 0.01 |
| 6 | Pleural Effusion | 1.63 | 1.49 | 1.78 | <0.001 |
| 7 | Pneumonia | 1.33 | 1.19 | 1.48 | <0.001 |
| 8 | Age at Report | 1.023 | 1.02 | 1.03 | <0.001 |
| 9 | Factor (Gender) M | 1.17 | 1.08 | 1.26 | <0.001 |
